# Impact and limitations of 3D computational modelling in transcatheter mitral valve replacement—a two-centre Dutch experience

**DOI:** 10.1007/s12471-024-01893-5

**Published:** 2024-09-16

**Authors:** Mark M. P. van den Dorpel, Mauricio Felippi de Sá Marchi, Zouhair Rahhab, Joris F. Ooms, Rik Adrichem, Sarah Verhemel, Claire Ben Ren, Rutger-Jan Nuis, Joost Daemen, Alexander Hirsch, Ben J. L. Van den Branden, Nicolas M. Van Mieghem

**Affiliations:** 1 Department of Cardiology, Cardiovascular Institute, Erasmus University Medical Centre, Rotterdam, The Netherlands; 2 Department of Cardiovascular Medicine, Heart Institute, Clinical Hospital, Faculty of Medicine, University of São Paulo, São Paulo, Brazil; 3 Department of Cardiology, Amphia Hospital, Breda, The Netherlands; 4 Department of Radiology and Nuclear Medicine, Erasmus University Medical Centre, Rotterdam, The Netherlands

**Keywords:** Transcatheter mitral valve replacement, Computational modelling, Multi-slice computed tomography, Left ventricular outflow tract obstruction

## Abstract

**Background:**

Transcatheter mitral valve replacement (TMVR) has emerged as a minimally invasive alternative to mitral valve surgery for patients at high or prohibitive operative risk. Prospective studies reported favourable outcomes in patients with annulus calcification (valve-in-mitral annulus calcification; ViMAC), failed annuloplasty ring (mitral valve-in-ring; MViR), and bioprosthetic mitral valve dysfunction (mitral valve-in-valve; MViV). Multi-slice computed tomography (MSCT)-derived 3D-modelling and simulations may provide complementary anatomical perspectives for TMVR planning.

**Aims:**

We aimed to illustrate the implementation of MSCT-derived modelling and simulations in the workup of TMVR for ViMAC, MViR, and MViV.

**Methods:**

For this retrospective study, we included all consecutive patients screened for TMVR and compared MSCT data, echocardiographic outcomes and clinical outcomes.

**Results:**

Sixteen out of 41 patients were treated with TMVR (ViMAC *n* = 9, MViR *n* = 3, MViV *n* = 4). Eleven patients were excluded for inappropriate sizing, 4 for anchoring issues and 10 for an unacceptable risk of left ventricular outflow tract obstruction (LVOTO) based on 3D modelling. There were 3 procedure-related deaths and 1 non-procedure-related cardiovascular death during 30 days of follow-up. LVOTO occurred in 3 ViMAC patients and 1 MViR patient, due to deeper valve implantation than planned in 3 patients, and anterior mitral leaflet displacement with recurrent basal septum thickening in 1 patient. TMVR significantly reduced mitral mean gradients as compared with baseline measurements (median mean gradient 9.5 (9.0–11.5) mm Hg before TMVR versus 5.0 (4.5–6.0) mm Hg after TMVR, *p* = 0.03). There was no residual mitral regurgitation at 30 days.

**Conclusion:**

MSCT-derived 3D modelling and simulation provide valuable anatomical insights for TMVR with transcatheter balloon expandable valves in ViMAC, MViR and MViV. Further planning iterations should target the persistent risk for neo-LVOTO.

**Supplementary Information:**

The online version of this article (10.1007/s12471-024-01893-5) contains supplementary material, which is available to authorized users.

## What’s new?

- Transcatheter mitral valve replacement (TMVR) is a minimally invasive alternative for patients at high or prohibitive operative risk for mitral valve surgery.
- Multi-slice computed tomography-derived 3D-modelling and TMVR simulation provide a comprehensive perspective of the spatial relationship between anatomical structures and the transcatheter heart valve.
- Left ventricular outflow tract obstruction remains an important challenge with TMVR.

## Introduction

Surgical repair or replacement is the standard of care for symptomatic severe mitral regurgitation or stenosis [1, 2]. Transcatheter mitral valve repair and replacement techniques have emerged as a minimally invasive alternative to surgery for patients at high or prohibitive operative risk. Mitral surgery for these indications may be challenging and even prohibitive because of anatomical constraints (e.g. excessive calcium that precludes safe debulking or a porcelain aorta), comorbidities, and/or frailty.

Apart from dedicated transcatheter mitral valve replacement (TMVR) devices, balloon-expandable transcatheter aortic valves are used as TMVR platforms in the context of native mitral valve disease with annulus calcification (valve-in-mitral annulus calcification; ViMAC) or a failing status after surgical mitral annuloplasty (mitral valve-in-ring; MViR) or bioprosthesis (mitral valve-in-valve; MViV) [3–8].

TMVR comes with specific anatomical challenges. Unlike the tube-shaped aortic valve, the mitral valvular apparatus is a complex, saddle-shaped structure that borders the left ventricular outflow tract (LVOT). Balloon expandable TMVR leverages the presence of mitral annulus calcification (MAC), a closed ring or a bioprosthesis for anchoring. A ‘neo-LVOT’ is created by the transcatheter valve frame that may cause obstruction through protrusion towards the interventricular septum and/or displacement of the (redundant and/or calcific) anterior mitral leaflet (AML) [9].

**Fig. 1:**
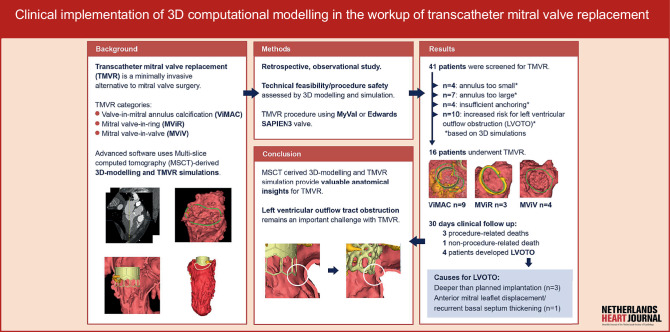
Infographic: Clinical implementation of 3D computational modelling in the workup of transcatheter mitral valve replacement

Multi-slice computed tomography (MSCT) complements TMVR planning [10]. Advanced software packages allow for MSCT-derived three-dimensional (3D) modelling of the anatomical substrate for digital and 3D printed TMVR simulations that offer unprecedented insights into the spatial relationships between the device and vital anatomical structures (Fig. 1). In this article, we describe the implementation and limitations of advanced MSCT-derived procedure modelling and simulation in the TMVR workup at two Dutch centres.

## Methods

### Patient selection

This retrospective study included all consecutive patients who were screened for TMVR in two centres (Erasmus MC, Rotterdam and Amphia Hospital, Breda, the Netherlands) between January 2018 and June 2023. Patients were discussed in a multi-disciplinary heart team consisting of interventional cardiologists, imagers, cardiac surgeons and geriatricians. MSCT-derived TMVR modelling and simulations were used to assess technical feasibility and procedure safety. All patients consented to the use of anonymous data for research purposes and proceedings were in accordance with the Declaration of Helsinki. The study did not fall under the scope of the Medical Research Involving Human Subjects Act per the Institutional Review Boards’ Review.

### TMVR simulation: step-wise approach

#### Image segmentation

**Fig. 2:**
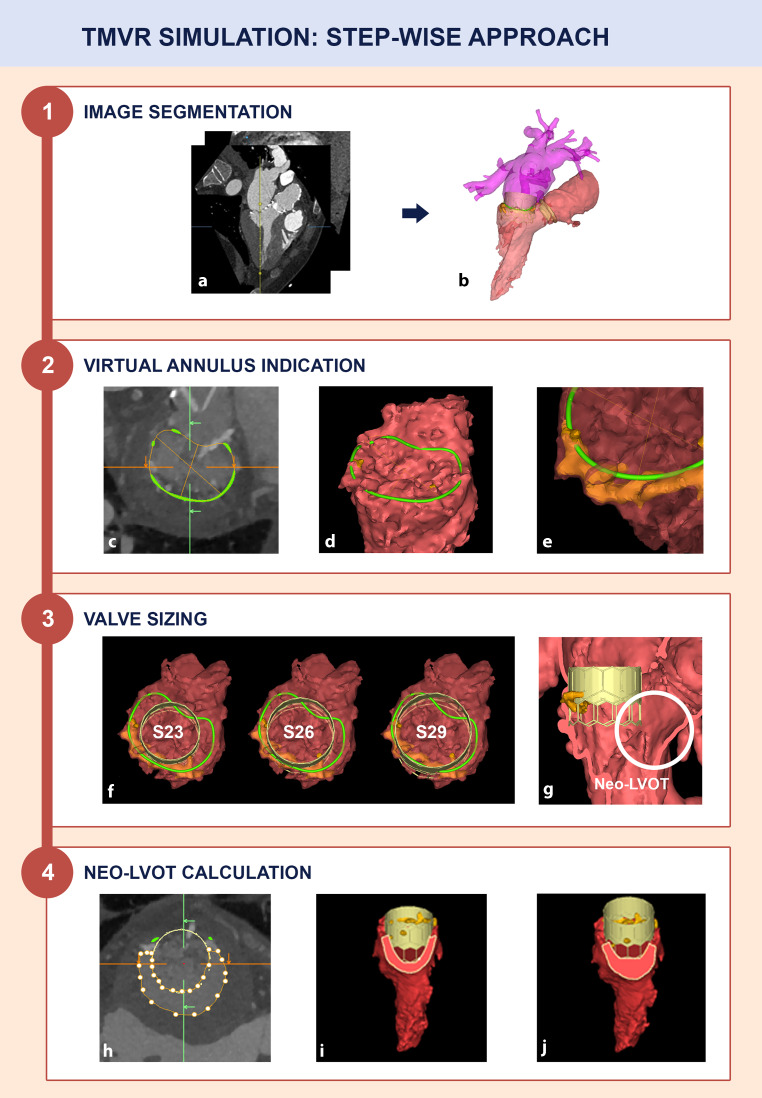
Transcatheter mitral valve replacement simulation flowchart. **a**, **b** 2D images were converted into a 3D model using computational modelling. **c** Annulus indication using 2D-planimetry. **d** Annulus indication using the 3D model. **e** Close-up of annulus indication in the presence of extensive annulus calcification. **f** Examination of different THV sizes within the virtual annulus (i.e. S23, S26 or S29). **g** Neo-left ventricular outflow tract (neo-LVOT). **h** Manual neo-LVOT area calculation using cross-sectional planimetry. **i**, **j** Automatic neo-LVOT area calculations based on the 3D model. Lower transcatheter heart valve implantation depth results in a smaller neo-LVOT area. *TMVR* transcatheter mitral valve replacement, *LVOT* left ventricular outflow tract, *THV* transcatheter heart valve

Full-cycle cardiac MSCT was used for analyses at different time points in the cardiac cycle. We used a dedicated semi-automated imaging segmentation software package (Mimics Enlight, Materialise, Leuven, Belgium) to create 3D anatomical models from small voxels (volumes of pixels) (Fig. 2a, b). The digital 3D model includes 3D visualisation of the major cardiac structures surrounding the mitral annulus and can be exported as a 3D printed model for enhanced understanding of the anatomy and benchtop device implantations.

#### Virtual annulus indication

The mitral annulus was identified using both 2D planimetry and by the placement of reference points directly on the 3D model and then served as a reference landing zone for the virtual transcatheter heart valve (THV) (Fig. 2c, d). The representation of the anatomy in the 3D model enhanced annulus detection even in cases displaying large pieces of calcification with distorted native annulus geometry (Fig. 2e).

#### Valve sizing

Maximum native mitral annulus size was measured at end-diastole [13]. TMVR procedures were simulated with different valve sizes to get enhanced insights into valve sealing and anchoring (Fig. 2f). Valve size was selected by annulus area and perimeter, according to cut-off values on the sizing chart of two balloon expandable valve platforms (MyVal THV system, Meril Lifesciences, Vapi, India or Edwards SAPIEN3 THV, Edwards LifeSciences Corp., Irvine, USA).

#### Neo-LVOT calculation

The ‘neo-LVOT’ was defined by the new LVOT anatomy after the TMVR procedure (Fig. 2g). The neo-LVOT area was simulated with various THV sizes and implantation depths (i.e. relative protrusion into the left ventricle) (Fig. 2h, i, j). An estimated neo-LVOT area at end-systole < 189 mm^2^ was previously identified as a strong predictor for LVOT obstruction (LVOTO), which is associated with procedural mortality and conversion to surgery. The accuracy of the predicted neo-LVOT has been confirmed in earlier studies that compared pre- and post-TMVR MSCT datasets [9, 11, 12]. Although the neo-LVOT area is smallest in the late systolic phase, most systolic volume is ejected in early to mid-systole [14, 15]. Additional neo-LVOT simulations throughout systole were provided to understand the dynamic neo-LVOT changes and maximise TMVR eligibility [13].

### TMVR procedure

**Fig. 3:**
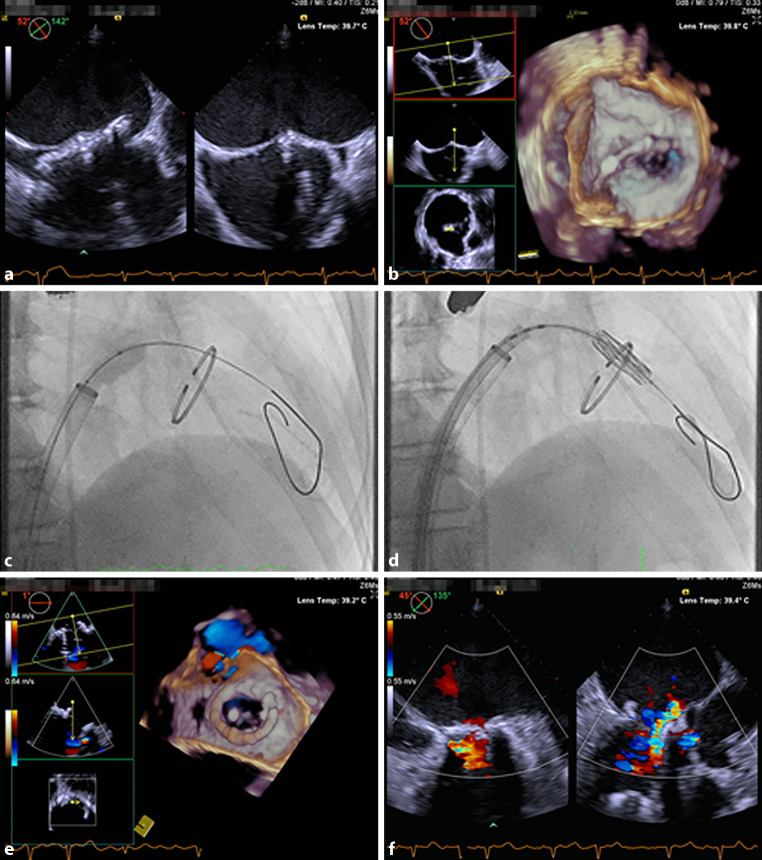
Transcatheter mitral valve replacement procedure. **a**, **b** Trans-septal puncture. **c** Trans-septal dilatation. **d** Balloon expandable transcatheter heart valve is deployed in mitral valve position. **e**, **f** Transoesophageal echocardiographic imaging confirms proper valve position and mild residual mitral regurgitation

TMVR procedures were performed under general anaesthesia and transoesophageal echocardiography guidance. A 14F to 18F sheath was introduced in the right femoral vein. After transseptal puncture and navigating through the left atrium and across the mitral valve a pre-shaped stiff wire (Safari, Boston Scientific, Marlborough, Massachusetts, USA) was positioned in the apex of the left ventricle (Fig. 3a, b).

The septostomy was dilated with a 14 mm balloon (Fig. 3c). A balloon-expandable transcatheter valve (MyVal THV, or Edwards SAPIEN3 THV) was then deployed under rapid pacing within the mitral annulus. Default implantation depth was aimed at LA:LV 30:70 or adjusted per MSCT simulations (Fig. 3d). To achieve the planned implantation depth, visualisation of anatomical landmarks through fluoroscopy and transoesophageal echocardiography was used. In MViV and MViR cases, the radiopaque annuloplasty or bioprosthetic ring was used for this purpose, while in ViMAC cases, CT segmentation of the calcification was used.

Bioprosthetic valve fracture (BVF) was not performed, but we hypothesise that this could potentially be considered in future MViV cases considering previous research in TAVI, which demonstrated improved echocardiographic gradients and increased valve area after BVF. At the same time, BVF may induce THV-leaflet injury and modify the neo-LVOT [14]. THV performance was evaluated by transoesophageal echocardiography and complemented by invasive transmitral and LVOT gradient assessment (Fig. 3e, f). Venotomy closure was achieved with suture-based closure devices (Perclose ProStyle, Abbott Vascular, Illinois, United States).

## Statistical analysis

Continuous variables are presented as median (25th–75th percentile) and categorical variables are expressed as numbers and percentages. Distributions of continuous variables were tested for normality by using the Kolmogorov-Smirnov test. Comparison of continuous variables between (sub)groups was performed using Mann-Whitney U and Kruskal-Wallis tests. Comparison of categorical variables between groups was performed with Chi-square tests. Analyses were performed using SPSS (27.0, IBM, Armonk, USA).

## Results

In total, 41 patients were screened for TMVR. Twenty-five patients were turned down for TMVR because of sizing issues (annulus too small in 4 patients and too large in 7 patients), insufficient anchoring (4 patients), or increased LVOTO risk (10 patients) based on 3D simulations. Sixteen patients were eligible for TMVR based on 3D modelling and underwent TMVR. Baseline demographics, echocardiographic characteristics and clinical outcomes of all patients are summarised in Table S1 of the Electronic Supplementary Material, and Tab. 1 and 2. Computed tomography derived computational modelling parameters are listed in Table S2 of the Electronic Supplementary Material. Among the patients who underwent TMVR, the median age was 73 (64–78) years and 44% of the patients were male. Indication for TMVR was mitral stenosis in 11 patients, mitral regurgitation in 3 patients, and mixed disease in 2 patients. Nine patients involved ViMAC cases, 3 MViR and 4 MViV. Median mitral valve mean gradients at baseline were similar for ViMAC (10.0 (9.0-12.0) mm Hg), MViR (9.0 (8.5–11.0) mm Hg) and MViV (9.5 (9.0–11.5) mm Hg, *p* = 0.30). Among the rejected patients, median age was 72 (61–81) years, 52% were male and STS risk scores were similar to the treated group (6.3 (3.1–7.7)% vs. 6.2 (3.4–7.1)%, *p* = 0.23). Baseline echocardiography demonstrated similar mitral mean gradients (1.7 (1.4–1.9) mm Hg vs. 1.6 (1.3–1.9) mm Hg, *p* = 0.19) and a higher left ventricular ejection fraction for the rejected patients (48 (38–57)% vs. 45 (40–55)%, *p* = 0.04) as compared with treated patients.

**Table 1:** Echocardiography data

	Baseline					30 days post TMVR			
	TMVR patients (*n* = 16)	ViMAC (*n* = 9)	MViR (*n* = 3)	MViV (*n* = 4)	Rejected patients (n = 25)	TMVR patients (*n* = 12)	ViMAC (*n* = 6)	MViR (*n* = 3)	MViV (*n* = 3)
LVEF (%)	45 (40–55)	50 (45–55)	40 (37–48)	41 (38–50)	48 (38–57)	47 (43–49)	49 (46–51)	43 (40–44)	45 (41–46)
MVA (cm^2^)	1.6 (1.3–1.9)	1.4 (1.2–1.7)	1.9 (1.8–2.1)	1.6 (1.5–1.8)	1.7 (1.4–1.9)	2.8 (2.2–3.0)	2.5 (2.1–2.9)	2.9 (2.9–3.1)	2.7 (2.4–2.9)
MV mean gradient (mm Hg)	9.5 (9.0–11.5)	10.0 (9.0–12.0)	9.0 (8.5–11.0)	9.5 (9.0–11.5)	9.0 (7.0–12.5)	5.0 (4.5–6.0)	5.5 (4.0–6.0)	4.5 (3.5–5.5)	5.0 (4.0–5.5)
LVOT mean gradient (mm Hg)	3.0 (2.5–4.0)	3.0 (2.5–4.0)	3.0 (3.0–3.8)	2.5 (1.5–3.0)	3.0 (2.5–4.5)	4.5 (3.5–4.5)	5.5 (5.0–6.0)	3.5 (3.0–3.5)	4.0 (3.5–4.5)
LVOT peak gradient (mm Hg)	6.0 (5.0–7.0)	6.0 (6.0–8.0)	5.0 (5.0–5.5)	4.5 (3.5–5.5)	5.5 (3.5–6.5)	7.5 (6.0–9.0)	8.5 (7.5–13.0)	6.0 (5.0–12.0)	6.0 (5.5–6.5)
LVOT peak gradient > 30 mm Hg	0 (0)	0 (0)	0 (0)	0 (0)	0 (0)	4 (33)	3 (50)	1 (33)	0 (0)
RVSP (mm Hg)	53 (44–62)	58 (48–63)	52 (44–59)	50 (39–64)	50 (41–62)	40 (29–50)	40 (31–54)	49 (46–51)	32 (26–38)
*MR severity*									
Grade 0	0 (0)	0 (0)	0 (0)	0 (0)	1 (4)	3 (25)	2 (33)	0 (0)	1 (33)
Grade 1	11 (69)	6 (67)	2 (67)	3 (75)	15 (60)	9 (75)	4 (67)	3 (100)	2 (67)
Grade 2	1 (6)	1 (11)	0 (0)	0 (0)	3 (12)	0 (0)	0 (0)	0 (0)	0 (0)
Grade 3	0 (0)	0 (0)	0 (0)	0 (0)	1 (4)	0 (0)	0 (0)	0 (0)	0 (0)
Grade 4	4 (25)	2 (22)	1 (33)	1 (25)	5 (20)	0 (0)	0 (0)	0 (0)	0 (0)
*MV pathology*									
Mitral stenosis	11 (69)	5 (56)	1 (33)	3 (75)	16 (64)	N/A			
Mitral regurgitation	3 (19)	2 (22)	1 (33)	1 (25)	5 (20)	N/A			
Mixed disease	2 (13)	2 (22)	1 (33)	0 (0)	4 (16)	N/A			

Values are median (25–75th percentile) or *n* (%).

*ViMAC* valve-in-mitral annulus calcification, *MViR* mitral valve-in-ring, *MViV* mitral valve-in-valve, *LVEF* left ventricular ejection fraction, *MVA* mitral valve area, *MV* mitral valve, *LVOT* left ventricular outflow tract, *RVSP* right ventricular systolic pressure, *MR* mitral regurgitation

**Table 2:** Clinical outcomes post-TMVR or post TMVR screening

	30 days post TMVR				30 days post TMVR screening	1 year post TMVR screening
	Total group (*n* = 16)	ViMAC (*n* = 9)	MViR (*n* = 3)	MViV (*n* = 4)	Rejected patients (*n* = 25)	Rejected patients (*n* = 24)
All-cause mortality	4 (25)	3 (33)	0 (0)	1 (25)	1 (4)	5 (21)
CV mortality	4 (25)	3 (33)	0 (0)	1 (25)	1 (4)	5 (21)
HF (re)admission	3 (19)	2 (22)	0 (0)	1 (25)	4 (16)	9 (38)
LVOT obstruction	4 (25)	3 (33)	1 (33)	0 (0)	N/A	N/A
> 2 residual MR	0 (0)	0 (0)	0 (0)	0 (0)	N/A	N/A
Pericardial effusion	2 (13)	1 (11)	0 (0)	1 (25)	0 (0)	0 (0)
Valve embolisation	0 (0)	0 (0)	0 (0)	0 (0)	N/A	N/A
Major vascular complication	1 (6)	0 (0)	1 (33)	0 (0)	N/A	N/A
Major bleeding complication	1 (6)	1 (11)	0 (0)	0 (0)	N/A	N/A
Stroke	0 (0)	0 (0)	0 (0)	0 (0)	0 (0)	1 (4)
Haemolytic anaemia	0 (0)	0 (0)	0 (0)	0 (0)	0 (0)	0 (0)
Surgical intervention after index procedure	2 (13)	1 (11)	1 (33)	0 (0)	N/A	N/A
Post-TMVR ASA	3 (19)	3 (33)	0 (0)	0 (0)	N/A	N/A
New pacemaker requirement	0 (0)	0 (0)	0 (0)	0 (0)	0 (0)	0 (0)
Valve thrombosis	0 (0)	0 (0)	0 (0)	0 (0)	0 (0)	0 (0)
Endocarditis	0 (0)	0 (0)	0 (0)	0 (0)	0 (0)	0 (0)
*NYHA class*						
NYHA I	4 (25)	1 (11)	1 (33)	2 (50)	0 (0)	0 (0)
NYHA II	5 (31)	2 (22)	2 (67)	1 (25)	4 (16)	1 (4)
NYHA III	3 (19)	3 (33)	0 (0)	0 (0)	18 (72)	14 (58)
NYHA IV	0 (0)	0 (0)	0 (0)	0 (0)	2 (8)	5 (21)
N/A	4 (25)	3 (33)	0 (0)	1 (25)	1 (4)	5 (21)

Values are median (25–75th percentile) or *n* (%).

*ViMAC* valve-in-mitral annulus calcification, *MViR* mitral valve-in-ring, *MViV* mitral valve-in-valve, *CV* cardiovascular, *HF* heart failure, *LVOT* left ventricular outflow tract, *MR* mitral regurgitation, *TMVR* transcatheter mitral valve replacement, *ASA* alcohol septal ablation, *N/A* not available

Four TMVR patients died within 30 days: 3 were procedure-related deaths and 1 involved a non-procedure-related cardiovascular death. One MViV patient died after atrial perforation, pericardial effusion and urgent surgery. One obese ViMAC patient with limited cardiac reserve died as a result of hypovolaemic shock due to clinically missed (covert) ongoing venous access site bleeding under full anticoagulation use. The third procedure-related death involved a frail ViMAC patient with a limited cardiac reserve who developed multi-organ failure due to cardiogenic shock shortly after TMVR. The last patient was a ViMAC patient who died from myocardial infarction 3 weeks post-procedure. Two ViMAC patients and 1 MViV patient needed readmission for heart failure. The duration of the re-admission was 7, 7 and 9 days. The mechanism of decompensation was found to be related to LVOTO in 1 ViMAC case. In the other ViMAC patient, no clear evidence for the decompensation mechanism was found other than pre-existing poor left ventricular function. The MViR patient requiring readmission displayed grade 2 mitral regurgitation at readmission, which was reported as grade 1 (and may have been underestimated) at the time of discharge.

**Fig. 4:**
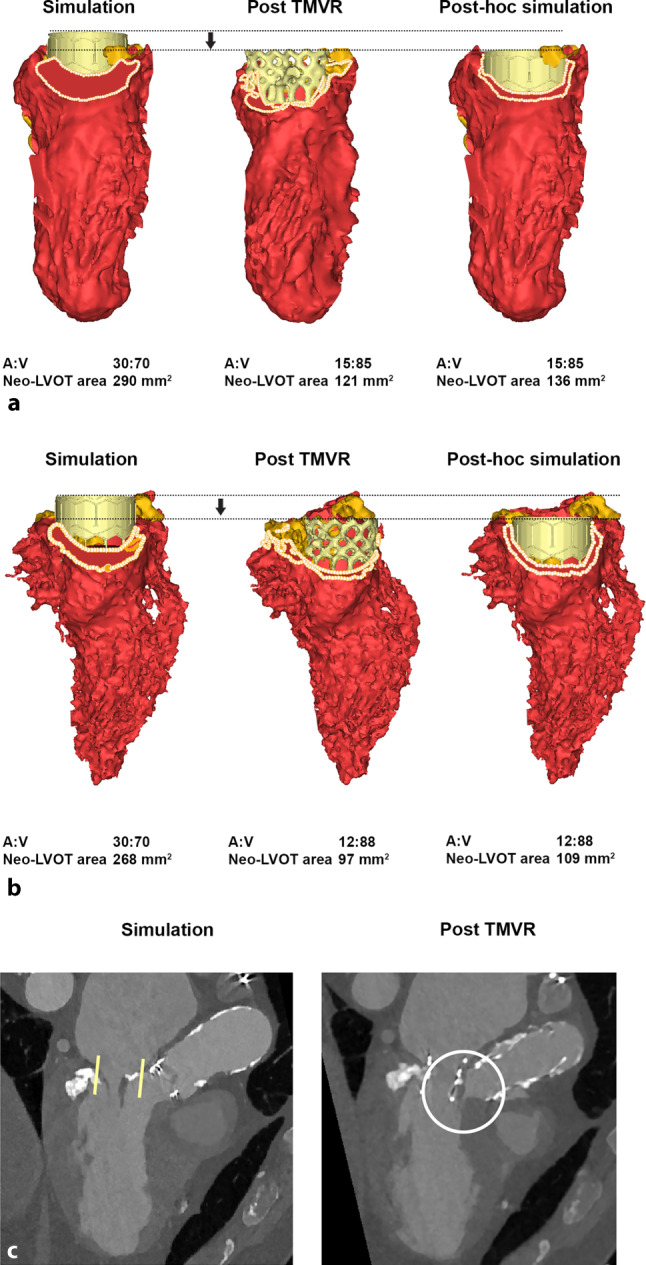
Pre-and post-procedural simulations in 3 patients who developed left ventricular outflow tract obstruction. **a** Left: Preprocedural simulation (ViMAC, SAPIEN3 26 mm THV, A:V 30:70) shows suitable neo-LVOT area. Yellow structures represent calcification. Middle: Postprocedural simulation displaying deeper (A:V 15:85) implantation and smaller neo-LVOT area. Right: Post hoc simulation using actual implantation depth (A:V 15:85) reproduces neo-LVOTO. **b** Left: Preprocedural simulation (ViMAC, SAPIEN3 2 6 mm THV, A:V 30:70) shows suitable neo-LVOT area. Middle: Postprocedural simulation displaying deeper (A:V 12:88) implantation and smaller neo-LVOT area. Right: Post hoc simulation using actual implantation depth (A:V 12:88) reproduces neo-LVOTO. **c** Left: Preprocedural 3‑chamber MSCT view shows suitable neo-LVOT area after ASA. Yellow bars indicate virtual THV implant position. Right: Postprocedural MSCT shows recurrent basal septum thickness and displacement of the anterior mitral leaflet (white circle), resulting in neo-LVOTO. *LVOT* left ventricular outflow tract, *neo-LVOTO* neo left ventricular outflow tract obstruction, *ASA* alcohol septal ablation, *THV* transcatheter heart valve, *MSCT* multi-slice computed tomography

LVOTO occurred in 4 patients (3 ViMAC, 1 MViR). In all cases, the simulated neo-LVOT area was well above the cutoff for LVOTO (i.e. > 189 mm^2^). In 3 patients, the THV was found to be implanted deeper than planned, resulting in a smaller than simulated neo-LVOT. Post-procedural MSCT was available in 2 of these patients and post hoc MSCT-derived 3D simulation using the actual valve implant depth was able to confirm the neo-LVOTO (Fig. 4a, b).

The fourth patient underwent alcohol septal ablation resulting in a thinner basal septum and larger, reassuring estimated neo-LVOT area as confirmed by MSCT prior to TMVR. However, post-TMVR MSCT revealed recurrent basal septum thickness and a narrow LVOT in combination with a substantial displacement of the AML (Fig. 4c). The 3 ViMAC patients underwent successful post-procedural alcohol septal ablation to resolve LVOTO, while the MViR patient eventually required surgical resection of the AML to resolve LVOTO.

There were no cases of valve embolisation, stroke, haemolytic anaemia, pacemaker requirement, valve thrombosis or endocarditis. The median mean mitral gradient after TMVR was 5.0 (4.5–6.0) mm Hg. There was no residual mitral regurgitation grade 2 or higher. Additional clinical examples of MViR, MViV and ViMAC 3D simulations are displayed in Fig. S1a–1c of the Electronic Supplementary (Material).

Among the rejected patients, five patients died within 1 year after TMVR screening. Nine patients needed admission for heart failure, with an average hospital stay of 8 days. Thirty days after screening, 20 of the rejected patients were in NYHA class III/IV, as compared with 3 TMVR patients.

## Discussion

In this case series, we used MSCT-derived 3D modelling and simulations for TMVR planning with transcatheter balloon-expandable valves in ViMAC, MViR, and MViV and found that: 1) many patients were deemed ineligible for TMVR based on 3D modelling and simulations because of issues related to sizing, anchoring or expected neo-LVOTO; 2) neo-LVOTO still occurred in one-fourth of patients despite advanced imaging planning, and 3 out of 4 cases occurred in ViMAC patients; 3) most neo-LVOTO cases resulted from a too deep valve implant depth and could be resolved with alcohol septal ablation; and 4) procedure-related mortality was substantial in this early experience, particularly with ViMAC.

Our observation of high procedure-related mortality with TMVR using transcatheter balloon expandable valves in ViMAC, MViR and MViV is consistent with previous registries. Clinical outcomes after TMVR are also highly variable among various patient categories. ViMAC emerged as a higher risk phenotype in our series. The MITRAL trial displayed a 30-day mortality of 21.8% with ViMAC, 11.5% with MViR and 8.1% with MViV [4]. Yoon et al. also found higher all-cause mortality after ViMAC (34.5%) compared with MViR (9.9%) and MViV (6.2%) at 30 days [7].

LVOTO was highest in our ViMAC group, which is concordant with previous studies, and has a multifactorial aetiology [9, 11]. Posterior MAC may preferentially pivot the transcatheter valve into an anterior direction towards the LVOT [15]. Furthermore, systolic anterior motion of the AML may contribute to LVOTO in these patients [16].

Four patients in our case series still developed LVOTO despite our simulations. There are several reasons why simulations may be inadequate [17]. First, this series included the early experience with simulations. The simulations were improved along the course of the study by doing more simulations per patient, including multiple transcatheter valve sizes and implant depths and at different systolic phases. However, digital simulations do not appreciate device/host interactions including heterogeneous MAC, frame deformation and frame or AML displacement. Finally, it may be difficult to accurately duplicate the simulated implantation depth in the cathlab. These intrinsic limitations of 3D simulation are relevant to all subgroups but appear more prominent with ViMAC as illustrated by the actual LVOTO rates that refuted the prior simulations.

In 2 patients with deeper than planned implantation who received a follow-up MSCT, the neo-LVOTO could be reproduced when the actual THV implantation depth was used. Therefore, more simulations including a greater variation of implant depths may be warranted to better appreciate the effects of deeper than intended THV implantations. Unexpected THV frame displacement and deformation also contributed to neo-LVOTO. Figure 4b illustrates how posterior MAC pushed the THV frame more anteriorly towards the LVOT than simulated.

Neo-LVOTO could be resolved by alcohol septal ablation in all but 1 patient in our study. This is consistent with previous research by Guerrero et al., which demonstrated that alcohol septal ablation is an effective and safe therapeutic option in TMVR-induced LVOTO [18]. Other percutaneous LVOT modification techniques have emerged over the last years (e.g. LAMPOON or SESAME) but were not applied in our centres [19, 20].

The high rate of screening failures due to anatomical causes and the relatively high procedure-related complication rate illustrate the need for further planning optimisation, dedicated transcatheter mitral valve platforms and ancillary techniques to enhance procedure safety in the context of ViMAC, MViR, and MViV.

Dedicated TMVR valve technologies are tested in ViMAC. The ViMAC arm of the Tendyne registry (Abbott Vascular, Illinois, United States), for instance, found a low rate of LVOTO (1 out of 20 patients) and complete elimination of mitral regurgitation in all patients [21]. Furthermore, the ENCIRCLE study (M3 platform, Edwards LifeSciences Corp., Irvine, USA) and the APOLLO study (Intrepid system, Medtronic, Minneapolis, USA) have also initiated ViMAC substudies. (Clinicaltrials.gov ID NCT04153292; NCT05496998). Nevertheless, LVOTO remains a significant risk and acknowledged reason for screen failure with all dedicated mitral valve technologies.

### Study limitations

The findings of these case series should be interpreted while taking into account the relatively small sample size and against the background of existing larger series that also included the early experience with 3D modelling and simulation for ViMAC, MViR and MViV.

## Conclusion

MSCT-derived 3D modelling and simulation provide valuable anatomical insights for TMVR with transcatheter balloon expandable valves in ViMAC, MViR and MViV. More than half of the TMVR candidates were rejected because 3D modelling and simulation identified inappropriate anatomy or an increased risk of neo-LVOTO. Further planning iterations should target the persistent risk for neo-LVOTO.

## Supplementary Information

Table S1 Baseline characteristics

Table S2 Computed tomography derived computational modeling parameters

Figure S1
