# Supplementary material for: Impact and limitations of 3D computational modelling in transcatheter mitral valve replacement—a two-centre Dutch experience
Source: Neth Heart J. 2024 Sep 16;32(12):442–54. doi: 10.1007/s12471-024-01893-5 (PMC11584822; doi:10.1007/s12471-024-01893-5)
Supplement: Supplementary file 1 — Table S1 Baseline characteristics [file 12471_2024_1893_MOESM1_ESM.docx]

**Table S1**. Baseline characteristics

|  |  |  |  |  |  |
| --- | --- | --- | --- | --- | --- |
|  | **TMVR patients (n=16)** | **ViMAC (n=9)** | **MViR (n=3)** | **MViV (n=4)** | **Rejected patients  (n=25)** |
| Age (years) | 73 (64 - 78) | 65  (61-73) | 73 (70-78) | 77  (73-83) | 72  (61-81) |
| Male | 7 (44) | 4 (44) | 2 (67) | 1 (25) | 13 (52) |
| STS score (%) | 6.2  (3.4-7.1) | 6.7 (3.1-8.3) | 5.2  (3.2-7.4) | 5.9 (3.6-10.7) | 6.3  (3.1-7.7) |
| Atrial fibrillation | 7 (44) | 1 (11) | 3 (100) | 3 (75) | 10 (40) |
| Diabetes | 5 (31) | 5 (55) | 0 (0) | 0 (0) | 6 (24) |
| COPD | 3 (19) | 2 (22) | 1 (33) | 0 (0) | 8 (32) |
| Renal failure | 6 (38) | 3 (33) | 2 (67) | 1 (25) | 7 (28) |
| Stroke | 4 (25) | 3 (33) | 1 (33) | 0 (0) | 8 (32) |
| Long-term anticoagulation | 13 (81) | 7 (78) | 3 (100) | 3 (75) | 18 (72) |
| CABG | 4 (25) | 2 (22) | 0 (0) | 2 (50) | 5 (20) |
| Prior AVR | 6 (38) | 5 (56) | 0 (0) | 1 (25) | 6 (24) |
| Prior TAVI | 3 (19) | 3 (33) | 0 (0) | 0 (0) | 5 (20) |
| Pre-emptive ASA | 2 (13) | 2 (22) | 0 (0) | 0 (0) | 0 (0) |
| Nyha class |  |  |  |  |  |
| nyha I | 1 (6) | 1 (11) | 0 (0) | 0 (0) | 0 (0) |
| nyha II | 2 (13) | 1 (11) | 0 (0) | 1 (25) | 5 (20) |
| nyha III | 12 (75) | 7 (78) | 2 (67) | 3 (75) | 17 (68) |
| nyha IV | 1 (6) | 0 (0) | 1 (33) | 0 (0) | 3 (12) |

Values are median (25-75th percentile) or n (%).

*ViMAC* valve-in-mitral annulus calcification; *MViR* mitral valve-in-ring; *MViV* mitral valve-in-valve; *COPD* chronic obstructive pulmonary disease; *AVR* aortic valve replacement; *TAVI* transcatheter aortic valve implantation; *ASA* alcohol septal ablation; *NYHA* New York Heart Association
