# Supplementary material for: Impact and limitations of 3D computational modelling in transcatheter mitral valve replacement—a two-centre Dutch experience
Source: Neth Heart J. 2024 Sep 16;32(12):442–54. doi: 10.1007/s12471-024-01893-5 (PMC11584822; doi:10.1007/s12471-024-01893-5)
Supplement: Supplementary file 2 — Table S2 Computed tomography derived computational modeling parameters [file 12471_2024_1893_MOESM2_ESM.docx]

**Table S2. Computed tomography derived computational modeling parameters**

|  |  |  |  |  |  |
| --- | --- | --- | --- | --- | --- |
|  | **TMVR patients (n=16)** | **ViMAC**  **(n=9)** | **MViR**  **(n=3)** | **MViV**  **(n=4)** | **Rejected patients  (n=25)** |
| Annulus area (mm^2^) | 553  (453-595) | 570  (505-620) | 540  (510-560) | 455 (440-475) | 545  (430-690) |
| <273 | 0 (0) | 0 (0) | 0 (0) | 0 (0) | 4 (16) |
| 273-345 | 0 (0) | 0 (0) | 0 (0) | 0 (0) | 1 (4) |
| 345-430 | 1 (6) | 1 (11) | 0 (0) | 0 (0) | 2 (8) |
| 430-546 | 7 (44) | 2 (22) | 1 (33) | 4 (100) | 5 (20) |
| 546-680 | 7 (44) | 5 (55) | 2 (66) | 0 (0) | 6 (24) |
| >680 | 1 (6) | 1 (11) | 0 (0) | 0 (0) | 7 (28) |
| Annulus perimeter (mm) | 82 (76-86) | 86 (79-90) | 82 (80-84) | 76 (74-77) | 81 (73-99) |
| Neo-LVOT area (mm^2^) | 408  (342–465) | 388  (318-483) | 349  (341-360) | 421  (404-481) | 334  (155-437) |
| High risk for LVOTO  (area <189mm^2^) | 0 (0) | 0 (0) | 0 (0) | 0 (0) | 10 (40) |
| Insufficient anchoring | 0 (0) | 0 (0) | 0 (0) | 0 (0) | 4 (16) |

Values are median (25-75th percentile) or n (%).
