# Supplementary figures and images for: Impact and limitations of 3D computational modelling in transcatheter mitral valve replacement—a two-centre Dutch experience
Source: Neth Heart J. 2024 Sep 16;32(12):442–54. doi: 10.1007/s12471-024-01893-5 (PMC11584822; doi:10.1007/s12471-024-01893-5)

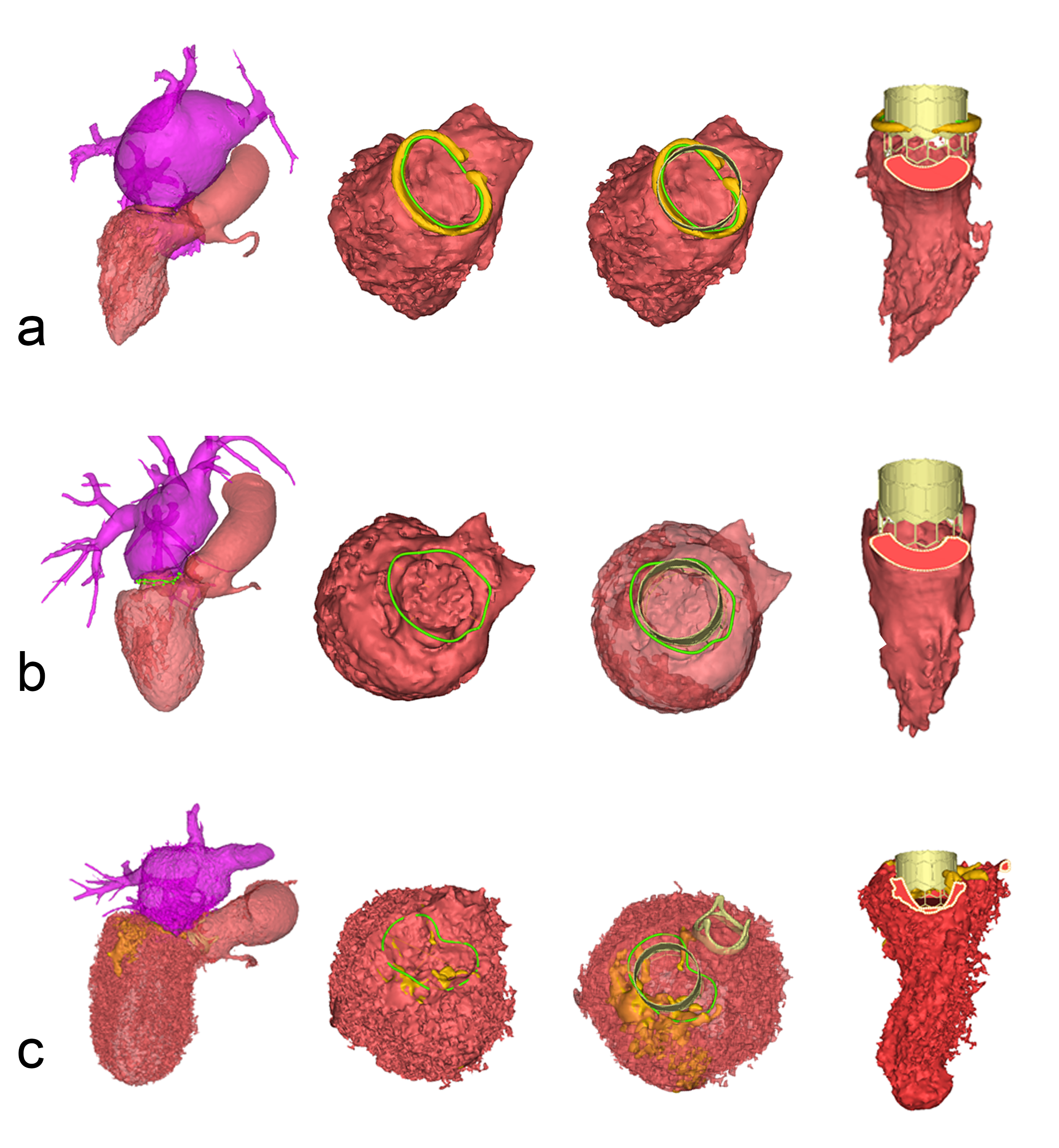

Supplement: Supplementary file 3 — Figure S1 [file 12471_2024_1893_MOESM3_ESM.tif]
